# Detection of Pathogens by a Novel User-Developed Broad-Range BR 16S PCR rRNA Polymerase Chain Reaction/Gene Sequencing Assay: Multiyear Experience in a Large Canadian Healthcare Zone

**DOI:** 10.3390/microorganisms14010240

**Published:** 2026-01-20

**Authors:** Thomas Griener, Barbara Chow, Deirdre Church

**Affiliations:** 1 Alberta Precision Laboratories, Diagnostic & Scientific Centre, Calgary, AB T2L 2K8, Canada; thomas.griener@albertaprecisionlabs.ca (T.G.); barbara.chow@albertaprecision.abs.ca (B.C.); 2 Department of Pathology & Laboratory Medicine, Cummings School of Medicine, University of Calgary, Calgary, AB T2N 1N4, Canada; 3 Department of Medicine, Cummings School of Medicine, University of Calgary, Calgary, AB T2N 1N4, Canada

**Keywords:** broad-range BR 16S PCR rDNA PCR, bacterial pathogen, molecular diagnosis, culture-negative, invasive infection

## Abstract

Between 2015 and 2022, we evaluated a novel broad-range (BR) 16S PCR rDNA PCR/Sanger sequencing assay to improve diagnosis of invasive infections in culture-negative specimens. Using dual-priming oligonucleotides (DPO), this assay analyzed ribosomal DNA from sterile fluids or tissues. A total of 762 specimens were analyzed from 661 patients: 61% had negative cultures and BR 16S PCR tests; 35% had negative cultures but positive BR 16S PCR tests; and only 4% had negative cultures with indeterminate BR 16S PCR results. After resolution of indeterminate BR 16S PCR results (i.e., 29 negative, 1 false-positive, and 1 positive) the assay showed a sensitivity of 98.26% (95% CI = 96.00–99.43%), specificity of 99.79% (95% CI: 99.82–99.99%), positive predictive value of 99.65% (95% CI: 97.56–99.95%), negative predictive value of 98.94% (95% CI: 97.51–99.55%), and accuracy of 99.21% (95% CI: 98.28–99.71%) for a disease prevalence of 38.10% (95% CI: 34.62–41.66%). Gram stain purulence predicted the BR 16S PCR result better (69.4%) than organisms (24.6%), but the latter had a higher PPV (78.5%). Increased peripheral WBC (86.1%) or CRP (71.8%) predicted positive BR 16S PCR results. Our DPO BR 16S PCR assay improved pathogen detection over culture and minimized contamination. Broad range 16S rDNA PCR/sequencing (BR 16S PCR) is an important diagnostic technique in cases with invasive infection due to fastidious or uncultivatable pathogens. However, appropriate case selection, the quality of clinical specimen, and the specific assay primers affect its performance. Our novel BR 16S PCR assay uses unique dual-priming oligonucleotides (DPO) primers and fast protocols for rapid, optimal detection of bacterial pathogens, while minimizing contamination. Fast BR 16S PCR assay reports occurred within 24–48 h. BR 16S PCR and culture analyzed a diverse range of clinical specimens from patients with invasive infections. BR 16S PCR demonstrated a high performance for accurately detecting pathogens, ruling out infections, and minimizing contamination. BR 16S PCR detection of a pathogen allowed the appropriate clinical management of one-third of patients in this cohort. BR 16S PCR is an essential tool for the clinical management of patients with invasive infection when primary cultures are negative or contaminated.

## 1. Introduction

Clinical management of serious invasive infections requires accurate organism identification, often to the species-level [1,2,3,4,5,6]. Accurate organism detection and identification not only allow for appropriately directed antimicrobial therapy but also selection of a more narrow-based antimicrobial regimen while improving duration of parenteral and stepdown oral therapy [7,8,9]. However, standard culture techniques may not yield a pathogen because the causative organism(s) are fastidious or uncultivatable or prolonged antibiotic courses of therapy are given prior to clinical specimen collection (i.e., suspected endocarditis and operatively harvesting heart valve tissue during delayed valve replacement) [10,11,12]. In addition, chronic infections involving biofilms, such as prosthetic joint infections (PJIs), present additional challenges for culture-based diagnosis, which may not yield a pathogen due to the sessile nature of these infections [13,14].

The use of molecular techniques to identify pathogens in clinical specimens has become increasingly prevalent, particularly in cases where patients present documented or confirmed invasive infections and traditional cultures yield negative results [15,16]. Among these molecular approaches, broad-range BR 16S PCR/sequencing has emerged as a valuable tool for the direct detection of bacterial genetic material from clinical samples, effectively bypassing the need for microbial culture growth [10,11,17,18,19,20]. For over twenty years, our lab has routinely used a unique in-house BR 16S rDNA PCR/sequencing (BR 16S PCR) assay with fast PCR protocols, using dual oligonucleotide primers to accurately identify bacterial isolates [3,4,6]. Building on this experience, we developed and published an evaluation of our team’s novel BR 16S PCR assay specifically designed to detect fastidious or uncultivatable bacterial organisms from excised heart valve tissue, thereby facilitating the diagnosis of culture-negative infectious endocarditis [11].

The present study details our subsequent experience applying our BR 16S PCR assay to a diverse array of sterile fluids (e.g., pericardial, synovial, cerebrospinal fluid, etc.) and tissues. Broad-range PCR/sequencing testing should be performed on select culture-negative clinical specimens based on set-out clinical and laboratory criteria, including rejection of non-sterile, highly contaminated specimens. Our laboratory implemented a formal scoring system to evaluate both clinical and laboratory criteria prior to approving this specialized test. BR 16S PCR was evaluated against culture in this study for its ability to diagnose various invasive infections, including those affecting the musculoskeletal system (MSK), cardiovascular system (CVR), and central nervous system (CNS), as well as other types of invasive infections. We compared the molecular assay results to clinically relevant diagnostic tests and reviewed clinical diagnoses, with particular attention to the outcomes and impacts on patient management.

## 2. Materials and Methods

### 2.1. Study Design

We performed a retrospective, cross-sectional study evaluating the effects of BR-PCR/sequencing on the diagnosis and management of infectious diseases in the Calgary Health Zone from the time of assay implementation for routine use in 2015 up to 2022. A total of 762 clinical specimens were analyzed from 661 patients for in-house molecular testing during this 7-year period. In patients who had multiple clinical specimens analyzed, results were scored once if all results agreed but scored individually if different results were obtained for each sample. Performance of all BR 16S PCR results were evaluated against results from clinical specimen Gram stains and cultures, patient test results for inflammation, comprising a peripheral white blood cell count (WBC) and C-reactive protein (CRP), and changes in clinical management brought about by the BR 16S PCR test result.

### 2.2. Patient Population

Medical Microbiologists enrolled patients at the request of the Infectious Diseases service if there was a high pre-test likelihood of having an invasive infection. As part of the enrollment, clinical cases were reviewed in detail with the attending physician to determine the patient’s biomarkers and the nature of the invasive infection, as outlined in detail in Section 2.3. Electronic medical records were also reviewed by the Medical Microbiologist for demographics and BR 16S PCR assay acceptance criteria, according to our scoring system, to assess the appropriateness of patients’ diagnoses and the quality of clinical specimens available for molecular analysis.

### 2.3. Laboratory Setting and Clinical Specimens

The Division of Microbiology, Alberta Precision Laboratories (APL), Diagnostic and Scientific Centre (DSC), and Calgary Zone performed microbiology testing. DSC is a large regional centralized laboratory that performs diagnostic testing for the entire Calgary Zone, including all ambulatory, hospitalized, and long-term care patients. Experienced physicians, surgeons, or interventional radiology collected the clinical specimens under sterile conditions during each procedure or operation. Fluids and tissues were immediately placed into a sterile container and promptly transported to the centralized laboratory within 2 h after collection. Clinical specimens were immediately processed and set-up for culture as outlined below. Residual specimen material storage occurred at temperatures ranging from −80 to −86 °C until DNA extraction.

Acceptance of clinical specimens for BR 16S PCR analysis was based on a direct request by the patient’s consulting Infectious Diseases specialist to the on-call Medical Microbiologist to determine if the clinical diagnosis was invasive and define how the microbial diagnosis impacted clinical management. Clinical specimens were sent for BR 16S PCR after a Medical Microbiologist’s review if at least one of the following patient criteria, and preferably both clinical specimen criteria, were present at the time of testing: (1) the Infectious Diseases service diagnosed the patient with an invasive infection, (2) the patient’s inflammatory biomarkers indicated the presence of active infection, (3) the available clinical specimen was sterile and of sufficient amount for BR 16S PCR testing, and (4) the Gram stain of the clinical specimen showed either polymorphonuclear cells (PMNs) and/or the presence of bacterial cells. Specifically, clinical specimens where the original Gram stain showed purulence and/or organisms and the patient had an elevated peripheral white blood cell count (WBC) that was a result of the neutrophils content and an elevated C-reactive protein (CRP) were preferred.

### 2.4. Microbiology Methods

#### 2.4.1. Culture

Standard methods were performed for blood and fluid cultures. Briefly, two separate sets of blood cultures [BacT/Alert FA (Aerobic) and FN (Anaerobic)], collected from adults, were immediately incubated in Virtuo instruments (bioMérieux, Laval, QC, Canada), and continuously monitored for up to 4 days for growth. Positive blood cultures were immediately removed, and an aliquot was spun into pellet before plating onto routine media, including Columbia blood agar (BA), chocolate agar (CHOC), MacConkey (MAC) agar, and Brucella blood agar (BBA) for isolation of aerobic and anaerobic bacteria. BA, CHOC, and MAC plates were aerobically incubated at 35 °C for up to 7 days, while the BBA plate underwent anaerobic incubation in an Anoxomat cabinet (Mart Microbiology Inc., Drachten, The Netherlands) for the same period. Similar methods were used to manage and plant sterile fluids. Aseptic disruption of tissues occurred in a grinder in 0.5 mL brain heart infusion (BHI) broth. The BHI tubes were centrifuged at 3000× *g* for 15 min, with discard of the supernatant. Aliquots (100 µL) of the prepared tissue sediment were immediately inoculated into a BacT/Alert FA and FN bottle, placed into the Virtuo cabinets for incubation, and continuously monitored for up to 7 days. A 20–30 µL drop of the sediment was planted onto BA, CHOC, MAC, and BBA plates for aerobic and anaerobic culture. Plates were incubated, as described earlier for positive blood cultures. Isolates were identified using a combination of matrix assisted laser-desorption ionization, time-of-flight mass spectrometry (MALDI-TOF MS, bioMérieux Canada, Inc., Saint-Laurent, QC, Canada), rapid phenotypic tests (Gram stain, catalase, coagulase, oxidase, indole), and BR 16S PCR whenever the initial MALDI-TOF MS result was discordant with phenotypic methods, gave a low discrimination result (i.e., ≤99.9%), or gave no result.

#### 2.4.2. Molecular Methods

Sterile fluids (minimum of 400 µL) were put in a sterile 1.5 mL microcentrifuge tube, centrifuged at 18,200× *g* for 5 min., and pellets were extracted after supernatant removal. Next, the pellets underwent the procedure outlined below. Tissues were minced with sterile scalpels and placed into a sterile 1.5 mL microcentrifuge tube. The QIAmp UCP Pathogen DNA Mini Kit protocol was used to extract DNA from the tissues (QIAGEN, Hilden, Germany). Briefly, 400 µL of digestion Buffer ATL and 40 µL of 20 mg/mL Proteinase K solution was used to resuspend the DNA extracted from the tissues. The samples were then briefly vortexed and incubated at 56 °C in a 1000 rpm Eppendorf thermomixer for a minimum of 1 h. Manufacturer’s instructions were used to further purify the DNA in the proteolytic digest. DNA was eluted into either 100 µL (sterile fluid specimens) or 150 µL (tissue specimens) of Buffer AVE. DNA purity was checked by measuring absorbance at 260 nm divided by 280 nm using a Nanodrop spectrophotometer to ensure an A260/280 ratio of 1.7 to 2.0. DNA eluate storage occurred at −20 °C until use. Fast BR 16S PCR was performed using dual priming oligonucleotide (DPO) primers that were purchased from Exiqon (Worburn, MA, USA) and used as previously described in references [11,21] with the Mastermix BR 16S PCR/18S Basic Kit (Molzym, Bremen, Germany). The PCR was set up in a 25 µL reaction volume with 5 µL of template DNA. An ABI Veriti thermocycler (Thermo Fisher—Life Technologies, Carlsbad, CA, USA) was used to perform standard PCR under the fast-thermocycling protocol, as previously published in reference [11]. All BR 16S PCR assays included the human beta globin gene as a positive internal control. A BLAST search against the Smart Gene Integrated Database Network System (IDNS 5 v3.11) (Lausanne, Switzerland) bacterial database indicated the most closely related species (http://www.Smartgene.com, accessed between 1 January 2015 to 30 December 2022) and the overall identify score compared to a well characterized reference sequence for all isolates was 99.9% with 0–2 mismatches, according to the CLSI MM-18 guideline [22]. Ripseq Sanger software (https://www.ripseq.com, accessed between 1 January 2015 to 30 December 2022) (Pathogenomix, Santa Cruz, CA, USA) was used to interpret mixed sequencing results.

### 2.5. Data Analysis

Standard descriptive statistical methods were used to perform data analyses. Performance of the BR 16S PCR assay results were compared to both conventional culture results and the patient’s clinical infectious diseases diagnosis. Sensitivity, specificity, positive predictive value (PPV), negative predictive value (NPV), and overall accuracy calculations provided a comprehensive evaluation of the assay’s diagnostic capabilities. Additionally, odds ratios and relative risks were determined for various laboratory tests and patient parameters to predict the likelihood of a positive BR 16S PCR result. These statistical measures helped clarify the association between specific clinical and laboratory findings and the outcomes of the molecular assay.

For classification purposes, a true positive occurred when at least one clinical specimen—either by culture or PCR sequencing—was positive in a patient with a documented clinical diagnosis of infection. A false-positive result occurred when a microorganism cultured in at least one clinical specimen, but the PCR was negative, and the patient did not have a confirmed clinical diagnosis. True negative results were those identified in clinical specimens from patients who exhibited no signs of infection, whether by culture or PCR. Conversely, a false-negative culture occurred when either PCR or culture was negative despite the patient having invasive infection. Indeterminate BR 16S PCR results were resolved as negative in most instances as this result agreed with culture negativity. Two instances where indeterminate BR 16S PCR detected a ‘possible’ pathogen or a contaminant resolved as positive and false-positive, respectively.

## 3. Results

### 3.1. Patient Characteristics

A total of 661 patients participated in the study. Most patients were adults (defined as individuals aged 14 years and older), accounting for 80.4% of the total cohort. The remaining participants were children and adolescents aged 14 years or younger, as detailed in Table 1. The overall mean age of the patient population was 41.8 years, with a standard deviation of 7.7 years. Within the adult subgroup, there was a higher proportion of males (56.5%) compared to females (43.5%), although there was no significant age difference between the male and female groups. Similarly, among pediatric patients, males comprised 60.0% of enrollments, while females accounted for 40.0%, with no significant age difference observed between these groups. In addition, no significant differences were found between patients with different types of invasive infection. Most patient assessments occurred either in the Emergency Department (ED) or during hospitalization. Few patients were ambulatory, and none resided in long-term care facilities.

Nearly all patients (98.6%) received antimicrobial therapy. The average duration of therapy among all patients was 9 days, with a standard deviation of 6.4 days. Patients diagnosed with cardiovascular-related (CVR) infections had the longest average duration of antibiotic therapy prior to undergoing surgery, at 15.4 days. In contrast, those with suspected septic arthritis or osteomyelitis received antibiotics for an average of 1.6 days before collection of clinical specimens. Patients with meningitis without extra-ventricular drain (EVD) insertion had cerebrospinal fluid (CSF) specimens collected in the Emergency Department before the initiation of antibiotic treatment.

### 3.2. Distribution of the Clinical Specimens

**Figure 1:**
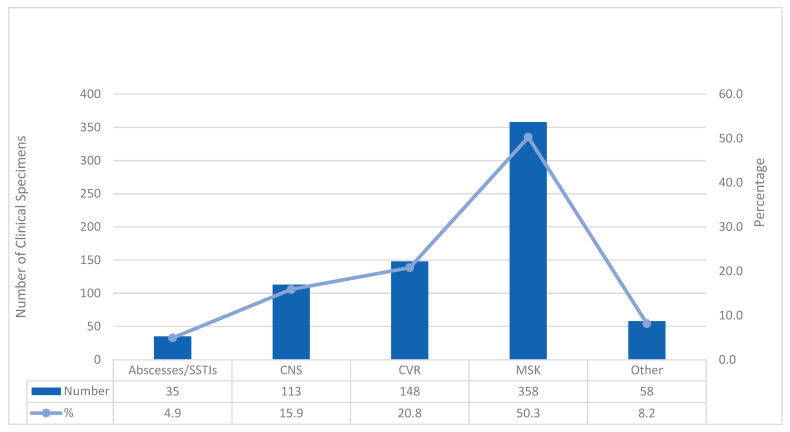
Distribution of the clinical specimens according to the primary site of infection. Definitions: (1) Skin and soft tissue infections (SSTIs), (2) musculoskeletal (MSK), comprising all synovial fluids and tissues from native and prosthetic joints and fluids and tissues from the spine, (3) cardiovascular (CVR), comprising heart valve tissue, pericardial fluids, and blood, (4) central nervous system, comprising cerebrospinal fluid (CSF), CSF from extra-ventricular drain (CSF-EVD), and brain fluids/tissues, and (5) Other, comprising clinical specimens of pleural fluids and lung tissues, peritoneal fluids, and bone marrow aspirates.

A total of 762 clinical specimens were analyzed; 81 (10.6%) patients had more than one tested specimen. Of the 101 specimens tested multiple times, 60 (58.4%) were second samples collected from the same infection site, while 51 (50.5%) consisted of two or more samples obtained from different sites of infection, with the number of specimens per case ranging from three to five. Figure 1 shows the distribution of 661 primary clinical specimens and 51 additional specimens from different infection sites by site of infection. Half of the specimens (358 cases) were collected from patients with suspected musculoskeletal (MSK) infections. Of these, 245 specimens (68.4%) came from cases of suspected septic monoarticular arthritis, distributed anatomically as follows: hip (62 cases, 25.3%), knee (150 cases, 61.2%), shoulder (8 cases, 3.3%), ankle (16 cases, 4.6%), elbow (4 cases, 1.6%), and wrist (5 cases, 2.0%). Another 64 specimens (17.9%) were from suspected prosthetic joint infections: total hip arthroplasty (16 cases, 25%), knee arthroplasty (39 cases, 61%), and shoulder arthroplasty (5 cases, 7.8%). A small number (4 cases, 1.1%) were linked to infected hardware following open reduction and internal fixation (ORIF) for long bone fractures. Lastly, suspected spinal infections accounted for 49 specimens (13.7%). These cases involved discitis, which may have occurred with or without vertebral osteomyelitis and/or paraspinal abscesses.

Cardiovascular (CVR) sites were the second most common clinical specimens, mainly due to infective endocarditis (IE) on native or prosthetic valves (132 of 148 cases, 89.1%). Few CVR blood cultures were analyzed (2 cases, 1.4%). Additional CVR infections included vascular graft infections (8 cases, 5.4%), intracardiac device or lead infections (3 cases, 2.0%), and pericarditis (3 cases, 2.0%).

Central nervous system (CNS) infections made up 113 cases (15.9%) out of the total. Of these, the majority—101 cases (89.3%)—were caused by meningitis or a combination of meningitis and ventriculitis, with twenty-one cases (20.8%) specifically connected to an implanted external–ventricular drain (CSF-EVD). Another twelve cases (17.5%) involved conditions like brain abscesses, cerebritis, or other types of brain lesions.

The study also reviewed 93 cases (13.1%) of suspected pleuropulmonary, peritoneal, and other abscess or SSTIs. Pleuropulmonary infections accounted for 44 cases (47.3%), including empyema and pulmonary lesions. Peritonitis was noted in 11 cases (11.8%)—mostly among dialysis patients—with culture-negative fluid samples. Other abscesses and SSTIs occurred in 35 cases (37.6%) across various sites such as liver, breast, limbs, scalp, catheter site, and sternum and included one necrotizing fasciitis case. Bone marrow specimens were tested in only three cases (3.2%).

### 3.3. Bacteria Detected by BR 16S PCR

BR 16S PCR identified 283 individual pathogens responsible for active invasive infections across various bacteria types: Gram-positive (203 cases, 71.7%), Gram-negative (59 cases, 20.8%), and a small subset of fastidious or unusual bacteria (8 cases, 2.8%). Mixed polymicrobial infections were observed in 13 cases (4.6%).

Among aerobic Gram-positive infections, approximately half were attributed to *Streptococcus* viridans group species, including *S. pneumoniae* [71 cases, 35%: 37 (52.1%) CVR cases, 16 (22.5%) empyema cases, 8 (11.3%) MSK cases, 9 (12.7%) CNS cases, and 1 (1.4%) peritonitis case in a patient receiving peritoneal dialysis]. *Enterococcus faecalis* accounted for seven cases (3.4%): six (85.7%) CVR cases and one (14.3%) MSK case. *Staphylococcus aureus* was detected in 27 cases (13.3%): 23 (85.2%) CVR cases, 3 (11.1%) MSK cases, and 1 (3.7%) abscess. Coagulase-negative *Staphylococcus* species, including *S. lugdenensis*, contributed to 30 cases (14.8%): 16 (53.3%) MSK, 11 (36.7%) CVR, and 2 (6.7%) SSTIs/abscesses. Beta-hemolytic *Streptococcus* species, including the *S. anginosus* group, comprised 43 cases (21.2%), spanning MSK (13, 30.2%), CVR (10, 23.3%), empyema (9, 21%), CNS (6, 14%), abscesses (2, 4.7%), and peritonitis (1, 2.3%). Other Gram-positive cocci (e.g., *Abiotrophia defectiva*, *Granulicatella adiacens*, *Gemella haemolysans*, *Aerococcus* spp.) were noted in eight cases (4%): four MSK (50%) and two CVR (25%) cases. Gram-positive bacilli (e.g., *Bacillus* spp., *Corynebacterium* spp., *Rothia* spp.) accounted for five cases (2.5%), all related to MSK or CVR infection.

Aerobic Gram-negative bacterial infections involved diverse organisms. Fastidious Gram-negative cocci (e.g., *Haemophilus* spp., *Kingella* spp., *Neisseria* spp., *Aggregatibacter aphrophilus*) caused 15 infections (36.6%) [6 (40%) empyema, 5 (33.3%) MSK, and 4 (26.7%) CNS cases]. Non-fermenters (e.g., *Acinetobacter* spp., *Burkholderia* spp., *Pseudomonas* spp.) were implicated in 14 infections (34.1%) [11 (78.6%) MSK, 3 (21.4%) CNS, and 1 peritonitis case]. *Enterobacterales* represented 12 infections (29.3%) [6 (50%) MSK, 4 (33.3%) abscess, 1 CNS, and 1 empyema].

Anaerobic bacteria were identified in 38 cases (13.4%), with Gram-positive species accounting for 20 (52.6%) and Gram-negative species for 18 (47.4%) cases. Anaerobic Gram-positive bacteria (e.g., *Cutibacterium acnes*, *Propionibacterium* spp., *Actinomyces* spp., *Clostridium* spp., and *Finegoldia magna*) were mainly associated with MSK infections (11 cases, 55%), followed by CVR (2, 10%), CNS (3, 15%), and abscesses (4, 20%). Anaerobic Gram-negative bacteria (e.g., *Bacteroides* spp., *Fusobacterium* spp., *Prevotella* spp., *Porphyromonas endodontalis*) predominantly caused MSK infections (12 cases, 66.7%), with additional CNS (3, 16.7%), empyema (1), and intra-abdominal abscess (1) cases.

Unusual or atypical infections exclusively detected by BR 16S PCR included a case of infective endocarditis attributed to either *Bartonella quintana* or *Tropheryma whipplei*, along with a blood sample positive for *Borrelia hermsii*. Additionally, skin and soft tissue infections (SSTIs) and abscesses were reported due to *Mycobacterium* or *Ureaplasma* species.

### 3.4. The Performance of BR 16S PCR Compared to Culture

Table 2 compares the BR 16S PCR assay to culture for primary clinical specimens (Table 2A) and other relevant samples from the same patient (Table 2B). In Table 2A, both methods matched pathogens in 35 cases, with one BR 16S PCR false negative, and agreed on 429 negative cultures. Both detected possible pathogens in three additional cases, while BR 16S PCR identified seven more true pathogen cases confirmed by clinical review. When culture grew contaminants (14 cases), BR 16S PCR found a true pathogen in six of those cases (42.9%). Of 31 indeterminate BR 16S results, most were negative (29), with one contaminant and one pathogen. BR 16S PCR showed higher sensitivity, specificity, and predictive value than culture, which had more false negatives and positives due to missed pathogens and contamination.

Table 2B presents a comparison of culture versus BR 16S PCR performance on other clinically relevant specimens, such as blood cultures and samples from various sites. In this group, about 41% of cases involved disease. Both methods detected the same pathogen in 45 cases, and they both agreed on 429 negative results. Culture identified a ‘possible’ pathogen in 19 additional cases, whereas BR 16S PCR detected possible pathogens in 15 cases. Of these, eight matched the organism found by culture, while seven involved different organisms; all of these were confirmed through clinical review.

When culture yielded contaminants (38 cases), BR 16S PCR found possible pathogens in 13 cases—one matching the culture’s organism, and twelve identifying different organisms, with all findings validated clinically. In most of these instances (12 out of 13, or 92%), the organisms detected by PCR differed from those found by culture. There was one instance where BR 16S PCR also identified a contaminant (*S. epidermidis* from a single valve tissue culture plate).

For indeterminate BR 16S results (30 cases), nearly all were resolved as negative (29 cases), with only one true pathogen detected, mirroring the culture results. Overall, BR 16S PCR showed similar diagnostic performance for other clinical specimens and demonstrated higher accuracy, sensitivity, specificity, and predictive value compared to culture for identifying infections or confirming their absence. Culture, on the other hand, continued to exhibit higher rates of false-negative and false-positive outcomes in these specimens, mainly due to undetected pathogens or contamination.

### 3.5. Prediction of Molecular Assay Results According to Clinical Specimen Microscopic Examination and Patient Biomarkers

Table 3, Table 4, Table 5 and Table 6 summarize how Gram stain results and patient biomarkers predict BR 16S PCR-sequencing outcomes. Purulence on Gram stain was more sensitive than detecting organisms for a positive BR 16S PCR result, but identifying organisms provided the highest overall sensitivity for pathogen detection. Elevated WBC and CRP were the most predictive biomarkers, though none showed sensitivity above 50% for PCR positivity. Gram stain organism positivity had the highest odds ratio (OR = 7.97) and relative risk (RR = 2.50), with other parameters presenting ORs between 2.0 and 3.0, and RRs between 1.5 and 2.0.

Table 4 summarizes an evaluation of musculoskeletal (MSK) infections, including culture-negative septic arthritis, periprosthetic joint infections (PJIs), and spinal discitis/osteomyelitis. Gram stain-detected purulence showed the highest sensitivity (77.5%) and was the best predictor of a positive BR 16S PCR result, compared to organism detection (7% sensitivity). The absence of organisms or PMNs on Gram stain strongly predicted negative PCR outcomes (NPV 87.0%). Elevated WBC and CRP were moderate predictors (about 60% predictive value). Purulence had the highest odds ratio (OR 2.95) and relative risk (RR 2.37), while a high PMN count had the lowest OR (0.54); other parameters ranged from OR 1.0 to 1.5 and RR 0.64 to 1.22.

Table 5 summarizes an evaluation of cardiovascular (CVR) cases, including culture-negative infective endocarditis or pericarditis, and vascular graft or ICD infections. Purulence on Gram stain (47.7%) and direct organism observation (22.73%) showed low sensitivity for predicting positive PCR outcomes, but their absence was highly specific for a negative result. Lack of WBC elevation had an NPV of 86.2% for ruling out infection before PCR testing. Elevated WBC and PMNs predicted positive PCR with over 95% sensitivity, while high PMN and CRP levels were about 80% predictive. Gram stain organism positivity had the highest odds ratio (OR = 10), followed by elevated WBC (OR = 7.07). Other parameters had moderate predictive power (3.0 < OR < 5.0). Relative risk (RR) was highest for elevated WBC (RR = 3.51), with other markers ranging from 1.62 to 2.22.

Table 6 summarizes an evaluation of CNS specimens, including CSFs from direct and EVD sources and brain tissues from culture-negative infections. Purulence on Gram stain was the most sensitive predictor of positive PCR (96%), while direct detection of organisms had lower sensitivity (56%). Absence of organisms on Gram stain was highly specific for negative PCR outcomes. In CSF, absence of PMNs had a high negative predictive value (97.6%) for ruling out infection. Increased WBC and PMN counts were both over 90% sensitive for positive PCR results, and elevated CRP and PMN counts showed moderate predictive value (>70% sensitivity). Odds ratios confirmed strong associations for purulence and organism positivity (ORs 26.67 and 47.09), with WBC and CRP elevations also notable (ORs 8–10). Relative risk analysis supported these findings, highlighting Gram stain purulence as the strongest predictor (RR 16.40).

## 4. Discussion

Our comprehensive, multiyear, regional assessment of a novel, rapid broad-range BR 16S PCR rDNA PCR/sequencing assay utilizing DPO primers represents the first clinical performance report of this unique approach. The study included testing hundreds of culture-negative specimens from patients with a diverse array of well-characterized invasive infections. Overall, the diagnostic performance of the BR 16S PCR assay demonstrated exceptionally high sensitivity, specificity, positive predictive value (PPV), negative predictive value (NPV), and accuracy relative to previously reported methods. The use of DPO primers contributed to a notably low contamination rate [21]. Furthermore, the implementation of fast PCR protocols enabled same-day result reporting in urgent cases. The findings confirm and build upon previous observations regarding the superior sensitivity of the rapid DPO BR 16S PCR assay in comparison to traditional culture, especially in the context of suspected invasive infections, particularly those related to MSK, CVR, and CNS sites [11]. Importantly, molecular identification of pathogens responsible for invasive infections led to changes and improvements in clinical management for roughly one-third of study patients, particularly those with suspected septic arthritis, prosthetic joint infections (PJIs), meningitis/ventriculitis or brain accesses, and infective endocarditis involving either native or prosthetic valves.

An important caveat of molecular testing in our study was rigorous review of the clinical case and selection of enrolled clinical specimens based on pre-set criteria (i.e., a standardized scoring system). This is essential to obtaining an accurate high-quality BR 16S PCR result that minimizes specimen contamination. Clinical specimens selected for BR 16S PCR analysis or tMGS analysis should be quality assured to optimize results; sterile fluids and tissues should have minimal prior handling and appropriate storage (refrigerated or frozen) before testing. However, few studies have addressed best practices for specimen collection and storage [23,24,25]. Our laboratory restricts requests for BR 16S PCR testing to Infectious Diseases physicians in consultation with a clinical microbiologist, who must approve the order based on the pre-test likelihood of improved pathogen detection, guided by Gram stain and inflammatory marker assessment. Our findings demonstrate that selecting specimens showing purulence and/or organisms on Gram stain, in patients with clinical signs of infection and elevated inflammatory markers, enhances the predictive sensitivity of the BR 16S PCR assay across various specimen types. Other investigators have also observed that a positive Gram stain is associated with 12-fold higher odds of a positive molecular result [26].

Earlier investigations into broad-range BR 16S PCR assays generally employed standard primers targeting either the V1–3 or V3–4 gene regions, with varied assay designs and outcomes depending on specimen type [12,13,27,28,29,30,31,32]. Notably, the highest concordance rates between culture and BR 16S PCR occurred with testing of heart valve tissues and joint fluids/tissues, with reported sensitivities ranging from 58% to 85%. Use of BR 16S PCR testing resulted in changes in clinical management for about 5% to 10% of patients in these studies. However, studies have reported lower sensitivity for BR 16S PCR followed by Sanger sequencing than for synovial fluid culture in diagnosing PJI [33]. A large-scale study assessed BR 16S PCR across 469 PJI specimens and 430 other sterile fluids/tissues that had also undergone culture [29]. Culture was positive for 170 (36.2%) of PJI specimens, but only 41.2% of these were PCR-positive. Additionally, 13 of 299 (4.3%) culture-negative PJI specimens were positive by PCR. For other sterile fluids and tissues, culture positivity was 12.1%, while PCR positivity was 61.5%. Furthermore, 31 of 378 (8.2%) culture-negative samples were positive by PCR alone. Another retrospective evaluation of 566 specimens from 460 patients found that 17% had positive molecular assay results, with clinical management changes in 5% of such cases [26]. Overall, BR 16S PCR/sequencing identified pathogens in 10% of culture-negative specimens, with the highest positivity in cardiovascular samples (37%) among clinically infected patients, whereas musculoskeletal specimens more frequently yielded positive cultures (*p* < 0.001) [26]. Prospective studies have also demonstrated the value of standard BR 16S PCR in enhancing pathogen detection in specific invasive infections.

Commercial BR 16S PCR assays have exhibited variable performance, with sensitivities ranging from about 60% to 95%. Examples include the Septifast (Roche) and UDM (Molyzym) assays, which have different configurations but few reported evaluations [18,34,35]. In a large prospective cohort involving 1370 clinical specimens (75 heart valves, 151 joint tissues, 230 synovial fluids, 840 whole blood, and 66 culture-negative CSF specimens), a commercial BR 16S PCR/sequencing assay demonstrated added value in 173 of 555 positive specimens (12.6%; 95% CI: 10.9–14.4), with the highest yields in heart valve and joint tissue samples. Blood cultures, however, had a high false-negative rate compared to culture, missing up to 7.1% of positive bloodstream infections [34].

More recently, next-generation sequencing (NGS) has emerged as a diagnostic tool for PJI, especially in culture-negative cases [36]. Although NGS is resource-intensive and costly for clinical laboratories [36], targeted metagenomic sequencing (tMGS) assays that use either Sanger of NGS techniques were recently developed for pathogen detection in sterile fluids and tissues [36]. Clinical studies have demonstrated similar or improved diagnostic performance for tMGS compared to culture, particularly for PJI and infectious endocarditis (IE) [16,26,37,38,39,40,41,42]. For example, a recent meta-analysis of seventy-nine studies compared the diagnostic accuracy of BR 16S rDNA PCR, multiplex PCR, and metagenomic next-generation sequencing (mNGS) in periprosthetic joint infections that included 3940 cases of prosthetic joint infection (PJI) and 4700 uninfected controls [42]. Although mNGS had the highest diagnostic odds ratio for identifying PJIs, the technical and practical challenges of these assays precluded its routine use in clinical laboratories [42]. Another study compared tMGS with a commercial shotgun metagenomics assay (Karius test) in blood and plasma from 34 suspected IE patients, yielding comparable detection rates [41]. The combined use of culture, Karius, and tMGS identified potential pathogens in 97% of ID cases, including five of the six cases with negative cultures.

There are important limitations to this study. It reflects the consolidated experience of a single centralized laboratory with longstanding expertise in molecular assay development. Results may not generalize to settings lacking such expertise in broad-range BR 16S PCR/sequencing. Additionally, Medical Microbiologists enrolled cases upon request of the Infectious Diseases service, but no patient chart reviews occurred. While this approach may have introduced selection bias, it restricted testing to appropriate culture-negative clinical specimens where the molecular assay would be beneficial to patient management.

## 5. Conclusions

Our novel BR 16S PCR assay improved detection for identifying underlying bacterial pathogens in a wide range of culture negative clinical specimens while having a low rate of contamination. Further study of our BR 16S PCR assay compared to a tMNGS assay would enhance evidence for the assay’s performance as a valuable diagnostic tool.

## Figures and Tables

**Table 1:** Characteristics of the patient population.

Parameter	Characteristic	N (%) ± SD
Number of subjects		662
Age, years (mean ± SD)		41.8 ± 7.7
Adults (≥14 yrs)		532 (80.4); 56.95 ± 10.8
	Male; Age, years (mean ± 2 SD)	300 (56.4); 56.5 ± 6.6
	Female; Age, years (mean ± 2 SD)	232 (43.6); 57.6 ± 4.5
Pediatrics (≤14 yrs)		130 (19.6); 6.3 ± 9.6
	Male; Age, years (mean ± 2 SD)	78 (60.0); 6.7 ± 7.4
	Female; Age, years (mean ± 2 SD)	52 (40.0); 6.6 ± 9.9
Location	Hospitalized or ED	589 (89)
	Ambulatory	33 (11)
Prior antibiotic therapy		652 (98.6)
Therapy prior to specimen collection		9 d ± 6.4 d

**Table 2:** (**A**) Performance of the BR 16S PCR compared to culture for detection of pathogens in the primary clinical specimens. (**B**) Performance of the BR 16S PCR compared to culture for detection of pathogens in the other clinical specimens.

(**A**)			
Culture Compared to BR 16S PCR ^a^			
	BR 16S PCR Result		
Culture Results	Positive	Negative	Total
Positive	35	10	45
Negative	251	448	709
Total	286	468	
BR 16S PCR Compared to Culture ^b^			
	Culture Result		
BR 16S Results	Positive	Negative	Total
Positive	283	1	284
Negative	5	467	472
Total	288	468	
(**B**)			
Culture Compared to BR 16S PCR ^a^			
	BR 16S PCR Result		
Culture Results	Positive	Negative	Total
Positive	45	15	60
Negative	240	404	664
Total	265	419	
BR 16S PCR Compared to Culture ^b^			
	Culture Result		
BR 16S Results	Positive	Negative	Total
Positive	283	1	284
Negative	5	467	472
Total	288	468	

(A): ^a^ Sensitivity [12.24% (95% CI = 8.67–16.61%)]; specificity [97.86% (95% CI: 96.11–98.97%)]; positive predictive value [88.78% (95% CI: 63.77–87.44%)]; negative predictive value [64.60% (95% CI: 63.56–65.63%)]; and accuracy [65.38% (95% CI: 61.87–68.78%)] for a disease prevalence of [37.93% (95% CI: 34.45–41.50%)]. ^b^ Sensitivity [98.26% (95% CI = 96.00–99.43%)]; specificity [99.79% (95% CI: 99.82–99.99%)]; positive predictive value [99.65% (95% CI: 97.56–99.95%)]; negative predictive value [98.94% (95% CI: 97.51–99.55%)]; and accuracy [99.21% (98.28–99.71%)] for a disease prevalence of [38.10% (95% CI: 34.62–41.66%)]. (B): ^a^ Sensitivity [15.79% (95% CI = 11.76–20.55%)]; specificity [96.42% (95% CI: 94.16–99.98%)]; positive predictive value [75.00% (95% CI: 63.04–84.07%)]; negative predictive value [62.73% (95% CI: 61.47–63.98%)]; and accuracy [63.78% (95% CI: 60.11–67.34%)] for a disease prevalence of 40.48% [95% CI: 36.83–44.21%]. ^b^ Sensitivity [98.94% (95% CI = 96.92–99.78%)]; specificity [100.00% (95% CI: 99.09–100.00%)]; positive predictive value [100.00% (95% CI: 98.69–100.00%)]; negative predictive value [99.26% (95% CI: 97.76–99.76%)]; and accuracy [99.56% (98.73–99.91%)] for a disease prevalence of 41.11% [95% CI: 37.40–44.89%].

**Table 3:** Prediction of the ability of the 16S assay to detect pathogen(s) in all clinical specimens according to microscopic examination and patient biomarkers.

All Specimens (N = 685)	Gram PMN (N = 685, 37.5%)	Gram Organism (N = 684, 37.6%)	WBC > 9.0 × 10^9^/L (N = 611, 37.8%)	Neutrophils HIGH (N = 351, 37.0%)	CRP > 50 mg/L (N = 420, 35.5%)
TRUE POSITIVE (Parameter predicts positive/16S positive)	175	62	198	73	107
FALSE POSITIVE (Parameter predicts positive/16S negative)	200	17	264	87	133
FALSE NEGATIVE (Parameter predicts negative/16S positive)	77	190	32	57	42
TRUE NEGATIVE (Parameter predicts negative/16S negative)	233	415	117	134	138
BR 16S PCR Assay Performance					
Sensitivity (correct prediction of positive)	69.4% (95% CI: 63.5–75.1%)	24.6% (95% CI: 19.4–30.4%)	86.1% (95% CI: 80.9–90.3%)	56.15% (95% CI: 47.18–64.84%)	71.8% (956% CI: 63.9–78.9%)
Specificity (correct prediction of negative)	53.8% (95% CI: 49.0–58.6%)	96.1% (95% CI: 93.8–97.7%)	30.7% (95% CI: 26.1–35.6%)	6.63% (95% CI: 53.86–67.12%)	50.9% (95% CI: 44.8–57.0%)
Positive Predictive Value	46.7% (95% CI: 43.4–49.9%)	78.5% (95% CI: 68.6–85.9%)	42.9%% (95% CI: 40.8–44.9%)	45.62% (95% CI: 40.16–51.20%)	44.6% (95% CI: 40.7–48.5%)
Negative Predictive Value	75.2% (95% CI: 71.1–78.8%)	68.6% (95% CI: 67.0–70.2%)	78.5% (95% CI: 71.9–83.9%)	70.16% (95% CI: 65.32–74.58%)	76.7% (95% CI: 71.3–81.3%)
Accuracy	59.6% (95% CI: 77.8–63.3%)	69.7% (95% CI: 66.1–73.2%	51.6% (95% CI: 47.5–55.6%)	59.0% (95% CI: 53.6–64.2%)	58.3% (95% CI: 53.5–63.1%)
Odds Ratio	2.65	7.97	2.74	1.97	2.64
Relative Risk	1.88	2.50	2.00	1.53	1.91

**Table 4:** Prediction of the ability of the 16S assay to detect pathogen(s) in musculoskeletal (MSK) specimens according to microscopic examination and patient biomarkers.

Bone and Joint Specimens (N = 309)	Gram PMN (N = 309, 23.0%)	Gram Organism (N = 309, 23.0%)	WBC > 9.0 × 10^9^/L (N = 274, 23.7%)	Neutrophils HIGH (N = 166, 28.9%)	CRP > 50 mg/L (N = 249, 25.3%)
TRUE POSITIVE (Parameter predicts positive/16S positive)	55	5	42	12	38
FALSE POSITIVE (Parameter predicts positive/16S negative)	128	13	131	45	103
FALSE NEGATIVE (Parameter predicts negative/16S positive)	16	66	23	36	25
TRUE NEGATIVE (Parameter predicts negative/16S negative)	110	225	78	73	83
BR 16S PCR Assay Performance					
Sensitivity (correct prediction of positive)	77.5% (95% CI: 66.0–86.5%)	7.0% (95% CI: 2.3–15.7%)	64.6% (95% CI: 51.8–76.1%)	25.0% (95% CI: 13.6–39.6%)	60.3% (95% CI: 47.2–72.4%)
Specificity (correct prediction of negative)	46.2% (95% CI: 39.8–52.8%)	94.5% (95% CI: 90.8–97.1%)	37.3% (95% CI: 30.8–44.4%)	61.9% (95% CI: 52.5–70.65%)	45.0% (95% CI: 37.4–52.1%)
Positive Predictive Value	30.1% (95% CI: 26.6–33.8%)	27.8% (95% CI: 12.4–51.0%)	24.3% (95% CI: 20.7–28.3%)	21.1% (95% CI: 13.4–31.4%)	27.0% (95% CI: 22.5–31.9%)
Negative Predictive Value	87.3% (95% CI: 81.4–91.5%)	77.3% (95% CI: 76.1–78.5%)	77.2% (95% CI: 70.0–83.1%)	67.0% (95% CI: 62.0–71.6%)	76.9% (95% CI: 70.2–82.4%)
Accuracy	53.4% (95% CI: 47.7–59.1%)	74.4% (95% CI: 69.1–79.2%)	43.0% (95% CI: 37.8–49.9%)	51.2% (95% CI: 43.3–59.0%)	48.6% (95% CI: 42.2–55.0%)
Odds Ratio	2.95	1.31	1.09	0.54	1.22
Relative Risk	2.37	1.22	1.07	0.64	1.16

**Table 5:** Prediction of the ability of the 16S Assay to detect pathogen(s) in cardiovascular (CVR) specimens according to microscopic examination and patient biomarkers.

CVR Specimens (N = 158)	Gram PMN (N = 158, 55.7%)	Gram Organism (N = 158, 55.7%)	WBC > 9.0 × 10^9^/L (N = 148, 56.1%)	Neutrophils HIGH (N = 59, 54.2%)	CRP > 50 mg/L (N = 47, 68.1%)
TRUE POSITIVE (Parameter predicts positive/16S positive)	42	20	82	26	26
FALSE POSITIVE (Parameter predicts positive/16S negative)	15	2	58	13	8
FALSE NEGATIVE (Parameter predicts negative/16S positive)	46	68	1	6	6
TRUE NEGATIVE (Parameter predicts negative/16S negative)	55	68	5	14	7
BR 16S PCR Assay Performance					
Sensitivity (correct prediction of positive)	47.7% (95% CI: 37.0–58.7%)	22.7% (95% CI: 14.5–32.9%)	98.8% (95% CI: 93.5–99.9%)	81.3% (95% CI: 63.6–92.8%)	81.3% (95% CI: 63.6–92.8%)
Specificity (correct prediction of negative)	78.6% (95% CI: 67.1–87.5%)	97.1% (95% CI: 90.1–99.7%)	7.9% (95% CI: 2.6–17.6%)	51.9% (95% CI: 32.9–71.3%)	46.7% (95% CI: 21.3–73.4%)
Positive Predictive Value	73.7% (95% CI: 63.0–82.2%)	90.9% (95% CI: 70.8–97.6%)	58.6% (95% CI: 56.7–60.4%)	66.7% (95% CI: 56.6–75.4%)	76.5% (95% CI: 66.3–84.3%)
Negative Predictive Value	54.5% (95% CI: 48.6–60.2%)	50.0% (95% CI: 47.0–53.0%)	83.3% (95% CI: 37.5–97.7%)	70.0% (95% CI: 60.0–84.0%)	53.9% (95% CI: 32.1–74.2%)
Accuracy	61.4% (95% CI: 53.3–69.0%)	55.7% (95% CI: 47.6–63.6%)	59.6% (95% CI: 51.2–67.6%)	67.8% (95% CI: 54.4–79.4%)	70.2% (95% CI: 55.1–82.7%)
Odds Ratio	3.35	10.00	7.07	4.67	3.79
Relative Risk	1.62	1.82	3.51	2.22	1.66

**Table 6:** Prediction of the ability of the 16S assay to detect pathogen(s) in central nervous system (CNS) specimens according to microscopic examination and patient biomarkers.

CSF Specimens (N = 101)	Gram PMN (N = 101, 24.8%)	Gram Organism (N = 101, 24.8%)	WBC > 9.0 × 10^9^/L (N = 95, 24.2%)	Neutrophils HIGH (N = 69, 21.7%)	CRP > 50 mg/L (N = 66, 22.7%)
TRUE POSITIVE (Parameter predicts positive/16S positive)	24	14	22	11	11
FALSE POSITIVE (Parameter predicts positive/16S negative)	36	2	49	19	13
FALSE NEGATIVE (Parameter predicts negative/16S positive)	1	11	1	4	4
TRUE NEGATIVE (Parameter predicts negative/16S negative)	40	74	23	35	38
BR 16S PCR Assay Performance					
Sensitivity (correct prediction of positive)	96% (95% CI: 79.7–99.9%)	56% (95% CI: 34.9–75.6%)	95.7% (95% CI: 78.1–99.9%)	73.3% (95% CI: 44.9–92.2%)	73.3% (95% CI: 44.9–92.2%)
Specificity (correct prediction of negative)	52.6% (95% CI: 40.8–64.2%)	97.4% (95% CI: 90.8–99.7%)	31.9% (95% CI: 21.4–44.0%)	64.8% (95% CI: 50.6–77.3%)	74.5% (95% CI: 60.4–85.7%)
Positive Predictive Value	40.0% (95% CI: 34.2–46.1%)	87.5% (95% CI: 63.1–96.6%)	31.0% (95% CI: 27.3–35.0%)	36.7% (95% CI: 26.5–48.2%)	45.8% (95% CI: 32.6–59.7%)
Negative Predictive Value	97.6% (95% CI: 85.3–99.6%)	87.1% (95% CI: 81.2–91.3%)	95.8% (95% CI: 76.7–99.4%)	89.7% (95% CI: 78.7–95.4%)	90.5% (95% CI: 80.2–95.7%)
Accuracy	63.4% (95% CI: 53.2–72.7%)	87.1% (95% CI: 79.0–93.0%)	47.4% (95% CI: 37.0–57.9%)	66.7% (95% CI: 54.3–77.6%)	74.2% (95% CI: 62.0–84.2%)
Odds Ratio	26.67	47.09	10.33	5.07	8.04
Relative Risk	16.40	6.76	7.44	3.58	4.81

## Data Availability

The data that supports the findings of this study are available from Alberta Health Services (AHS), Alberta Precision Laboratories (APL) (formerly Calgary Laboratory Services) but restrictions apply to the availability to these data, which were used under the ethics agreement for the current study and so are not publicly available. Data are, however, available from the author upon reasonable request and with permission of AHS/APL.

## Abbreviations

This manuscript uses the following abbreviations:

BR 16S PCR	Broad range BR 16S PCR rDNA polymerase chain reaction and Sanger sequencing
BR 16S PCR rDNA gene	BR 16S PCR ribosomal ribonucleic acid gene
DNA	deoxyribonucleic acid
APL	Alberta precision laboratories
DSC	Diagnostic and Scientific Centre
BA	blood agar
BBA	Brucella blood agar
CHOC	chocolate agar
MAC	MacConkey agar
MALDI-TOF MS	matrix assisted laser desorption-time of flight mass spectrometry
MSK	musculoskeletal
PJI	prosthetic joint infection
CVR	cardiovascular
CNS	central nervous system
CSF	cerebrospinal fluid
SSTI	skin and soft tissue infection
CRP	C-reactive protein
WBC	white blood cell count
PMN	polymorphonuclear
PPV	positive predictive value
NPV	negative prediction value
OR	odds ratio
RR	relative risk
tMGS	targeted metagenomics
Ct	cycle threshold
Sterile fluids	pericardial, peritoneal, synovial, cerebrospinal fluid
